# Reviewers in 2020

**DOI:** 10.1098/rsob.210040

**Published:** 2021-04-07

**Authors:** Jon Pines

**Affiliations:** *Editor-in-Chief*

The Editors of *Open Biology* would like to thank all reviewers who contributed their time by refereeing manuscripts submitted throughout 2020. From previous feedback, we know that most reviewers value recognition of their efforts by the journal and through services, such as Publons, which is integrated with *Open Biology*.

To provide an opportunity for recognition, we list the names of all reviewers who opted in. This article is made permanent and citeable by its digital object identifier (DOI). We encourage you to quote your contribution in your applications for tenure and grants or other forms of research assessment. Where you have provided it, we have included your ORCID, so your contribution can be unambiguously assigned to you.

The team really does appreciate all the work our reviewers do on behalf of the journal, and we look forward to working with you again in 2020.

Abramov A
Adamska M
Ahmad K https://orcid.org/0000-0002-1095-8445
Ahmed M
Allen A
Allen-Vercoe E
Archambault V https://orcid.org/0000-0002-2857-7667
Arulanandam J
Asgari S
Avidor-Reiss T https://orcid.org/0000-0003-0918-526X
Baena-Lopez LA
Barnett S
Benanti J
Ben-David U
Ben-Zvi A
Bidmon H
Black BE https://orcid.org/0000-0002-3707-9483
Blankesteijn M
Blewitt M
Bossis G
Boucher D
Bradley W
Bronner M
Brunet F
Bultynck G
Cantu C
Carter C
Cellini B
Chacińska A
Chan D
Chan K-l https://orcid.org/0000-0002-8796-8385
Chao J https://orcid.org/0000-0002-5895-9156
Charras G
Chen R-H
Cheung M
Chisholm A
Chitnis A
Chowdary TK
Cimini D
Ciulli A https://orcid.org/0000-0002-8654-1670
Conduit P https://orcid.org/0000-0002-7822-1191
Consonni FM
Coombs M
Cooper C
Cornelis P
Craig E
Cromer M
Daly AF
Dandekar AA
D'Angiolella V
Dao Thi L
Davey P
Dessi' D https://orcid.org/0000-0001-5713-741X
Dessimoz C
Dingli D
Draviam VM https://orcid.org/0000-0001-8295-3689
Duennwald ML
Dutcher S https://orcid.org/0000-0001-5689-5753
Duzgunes N
Dwivedi VP
Eichler K https://orcid.org/0000-0002-7833-8621
Eschbach C
Estepar RSJ
Fairlamb A
Faso C https://orcid.org/0000-0002-1831-9365
Ferenbach D
Forth J-H
Francis-West P
Gall C
Gardner D
Gartner A
Gemma M https://orcid.org/0000-0001-7941-983X
Gerber B https://orcid.org/0000-0003-3003-0051
Giansanti MG http://orcid.org/0000-0002-6753-7262
Ginsberg D
Gioeli D
Goldsmith M
Gossmann T
Grabowska A
Graham A
Gratchev A
Graves L
Groenen M
Gruszecki W
Gryder B
Guttery D
Guzzo M
Hadjivasiliou Z https://orcid.org/0000-0003-1174-1421
Hammarton T
Hamre K
Hannon G http://orcid.org/0000-0003-4021-3898
Hasnain S https://orcid.org/0000-0002-3269-1518
Helgason GV https://orcid.org/0000-0003-1616-132X
Hellmann N
Hengst U
Hiraoka Y
Hoffmann FG
Holland A
Hubmacher D
Izumi Y
Jacobson A https://orcid.org/0000-0002-5661-3821
Jain A
Jakimowiz D https://orcid.org/0000-0002-7857-064X
James L
Janssen J
Jeevaratnam K https://orcid.org/0000-0002-6232-388X
Jia S
Johnson M
Jorgensen H
Jurak I
Karch F https://orcid.org/0000-0003-4722-5170
Keren N
Kim S
Kitagawa D
Kojima T https://orcid.org/0000-0001-9949-1943
Konishi M
Krebs J
Kuan Y-S
Kurita T https://orcid.org/0000-0003-4281-5884
Lang BF
Laxman S https://orcid.org/0000-0002-0861-5080
Layne M
Lee H-J
Leisegang K
Li C-j https://orcid.org/0000-0003-1389-9820
Liang B
Lie C
Ling E
Lorenz A
Lou E
Lum L
Ma D
Maccioni R
MacKenzie L
maiato h
Malecki M
Mansfeld J http://orcid.org/0000-0002-0562-8206
Manzanares M
Marchuk D https://orcid.org/0000-0002-3110-6671
Marshall E
Masai H
Mason R
Mata J
Maurer S https://orcid.org/0000-0003-4532-1994
Mayer M
Mayor S
Megraw T
Metzen E
Metzger M
Milan M
Mingarro I https://orcid.org/0000-0002-1910-1229
Minna J
Moreira P
Mottram J
Muneoka K
Munsky B
Murdock C https://orcid.org/0000-0001-5966-1514
Musacchio A
Nabavi SM https://orcid.org/0000-0001-8859-5675
Nagel K
Nantasenamat C
Nardelli P
Netherton C
Nichols S
Nonaka S
Okamoto N
Olivares Reyes JA
Orzack S
Panfilio K https://orcid.org/0000-0002-6417-251X
Parchem R
Park J
Pavlidis P https://orcid.org/0000-0002-0426-5028
Pecorino L
Pereira G
Perez-Chacon G
Polacek N
Pruyne D https://orcid.org/0000-0002-2873-0035
Rabinowitz J
Ranjan T
Rass U
Reymann A-C
Rhind N
Robinson S
Romano A https://orcid.org/0000-0003-2766-8847
Rossi F
Roué G https://orcid.org/0000-0003-0245-2257
Rowley C https://orcid.org/0000-0001-9107-6972
Saavedra E
Sage P
Sandovici I
Sarinay-Cenik E
Schlosser G
Scholpp S
Schuhmacher J
Schultheiss T
Sengupta S
Shanker A
Shi X https://orcid.org/0000-0001-5242-8189
Shih Y-L
Shim J
Si K
Singh P
Smith A https://orcid.org/0000-0001-5628-7336
Smith B
Soto-Rifo R https://orcid.org/0000-0003-0945-2970
Storchova Z
Struhl G
Su C-Y
Suzanne M
Swan A
Swann K
Takahashi T
Tanaka K
Tandon R
Tarantola M
Tercero JA
Terret M-E
Tikhonova M
Todo T
Ulrich S
Uttaro A
Velikova T
Voisine C
Walejko J
Walker D
Wang C https://orcid.org/0000-0003-1477-1466
Wang F https://orcid.org/0000-0001-8730-0003
Wang L https://orcid.org/0000-0002-1049-9170
Wang Q
Wang Y
Ward H
Wei Q https://orcid.org/0000-0003-1214-010X
Weil D https://orcid.org/0000-0001-7630-1772
Weissman K
Welburn J https://orcid.org/0000-0002-5440-6060
West S
Westermann S
Wilcockson D
Wilgus T
Winklhofer K
Wodarz A
Wodarz D https://orcid.org/0000-0002-8017-3707
Wolfner M
Wu Y
Xiao H
Xu J https://orcid.org/0000-0003-1910-4603
Xu P https://orcid.org/0000-0002-7318-8548
Yan F
Yan J https://orcid.org/0000-0002-1303-1952
Yang X
Ylla G
Yun M
Zhang B
Zhang R
Zhou X https://orcid.org/0000-0001-5721-944X
Zinn K
Zou Z

